# Reduced Serum Pannexin-1 Levels in Obstructive Sleep Apnea and Their Association with Nocturnal Hypoxemic Burden

**DOI:** 10.3390/jcm15114299

**Published:** 2026-06-02

**Authors:** Esma Tuğba Canlı, Önder Öztürk, Hilal Türkmen Kaya, Fevziye Burcu Şirin, Doğukan Gümüşcan, Tutku Aydın, Adnan Karaibrahimoğlu

**Affiliations:** 1 Department of Chest Diseases, Faculty of Medicine, Süleyman Demirel University, 32260 Isparta, Türkiye; dronderozturk@gmail.com (Ö.Ö.); paradiesehilal1995@gmail.com (H.T.K.); tutkuaydin1994@gmail.com (T.A.); 2 Department of Medical Biochemistry, Faculty of Medicine, Süleyman Demirel University, 32260 Isparta, Türkiye; fbsirin@gmail.com (F.B.Ş.); dogukangumuscan97@gmail.com (D.G.); 3 Department of Biostatistics and Medical Informatics, Faculty of Medicine, Süleyman Demirel University, 32260 Isparta, Türkiye; adnankaraibrahim@gmail.com

**Keywords:** obstructive sleep apnea, pannexin-1, nocturnal hypoxemia, hypoxic burden, sleep biomarkers, polysomnography

## Abstract

**Background**: Obstructive sleep apnea (OSA) is a common sleep-related breathing disorder characterized by recurrent episodes of intermittent hypoxia and systemic inflammation. Pannexin-1 (Panx1) is a transmembrane channel involved in ATP release and purinergic signaling and has been implicated in hypoxia-related inflammatory responses. However, the clinical relevance of circulating Panx1 levels in patients with OSA remains poorly understood. This study aimed to evaluate serum Panx1 concentrations in patients with OSA and to investigate their association with nocturnal hypoxemic burden. **Methods**: In this cross-sectional study, 40 patients with obstructive sleep apnea (OSA) and 40 control subjects underwent overnight polysomnography for diagnostic evaluation. Serum Panx1 concentrations were measured using an enzyme-linked immunosorbent assay (ELISA). Logistic regression models were constructed to evaluate the association between Panx1 and OSA status while adjusting for clinical covariates. In addition, a propensity score–matched sensitivity analysis based on age, sex, and body mass index was performed to further assess potential confounding. Receiver operating characteristic (ROC) curve analysis was performed to evaluate the diagnostic performance of Panx1 alone and in combination with clinical variables. **Results**: Serum Panx1 levels were significantly lower in patients with OSA than in controls (4.27 ± 2.66 vs. 6.24 ± 4.75 ng/mL, *p* = 0.013). Although Panx1 was not an independent predictor of OSA after adjustment for age, sex, and body mass index, its integration with clinical variables significantly improved diagnostic discrimination. The area under the receiver operating characteristic curve increased from 0.662 for Panx1 alone to 0.858 in the fully adjusted model. Sensitivity analyses attenuated the observed association after matching for major baseline characteristics, suggesting a potential contribution of demographic and anthropometric factors. In addition, Panx1 concentrations were inversely correlated with markers of nocturnal hypoxemic burden, particularly the cumulative time spent with oxygen saturation below 90% (T90). **Conclusions**: Lower serum Panx1 concentrations were associated with OSA status and nocturnal hypoxemic burden. While Panx1 alone demonstrated modest discriminatory ability, its integration with established clinical factors improved diagnostic performance. These findings suggest that Panx1 may represent a biologically plausible adjunct biomarker reflecting hypoxic burden and may contribute to multi-parameter approaches for OSA risk assessment; however, further validation in larger matched cohorts is warranted.

## 1. Introduction

Obstructive sleep apnea (OSA) is a prevalent sleep-related breathing disorder characterized by repetitive collapse of the upper airway, leading to chronic intermittent hypoxia (IH), hypercapnia, and sleep fragmentation [1]. The physiological stress induced by IH triggers a cascade of pathological processes, including systemic inflammation, oxidative stress, and sympathetic overactivity, which collectively contribute to increased cardiovascular and metabolic morbidity [2]. Despite the high prevalence of OSA, the molecular mechanisms underlying its systemic manifestations remain incompletely understood, necessitating the identification of novel biomarkers that reflect cellular stress and inflammatory signaling.

Pannexin-1 (Panx1) has recently emerged as a critical mediator in the context of sterile inflammation and cellular response to hypoxia [3]. As a large-pore transmembrane channel, Panx1 facilitates the release of “find-me” signals, most notably adenosine triphosphate (ATP), from stressed or apoptotic cells into the extracellular space [4]. This purinergic signaling pathway plays a pivotal role in activating the NLRP3 inflammasome and modulating the recruitment of inflammatory cells [5]. Given that intermittent hypoxia is a hallmark of OSA, it is plausible that Panx1-mediated ATP release serves as a key link between nocturnal respiratory events and systemic inflammatory responses.

Recent experimental evidence suggests that Panx1 expression and function may be sensitive to oxygen fluctuations and hypoxia-related cellular stress. Chronic or recurrent hypoxic exposure has been shown to induce inflammatory and stress-related signaling pathways, which may influence Panx1 regulation over time [6,7]. Given that chronic intermittent hypoxia is a central feature of OSA, such mechanisms may contribute to alterations in circulating Panx1 concentrations in affected individuals. Furthermore, Panx1 has been implicated in the regulation of vascular tone and hypoxia-related vascular responses, both of which are relevant to the systemic complications of OSA [7,8]. However, clinical data investigating circulating Panx1 concentrations in patients with OSA remain remarkably scarce.

In this study, we hypothesized that serum Panx1 levels would be altered in patients with OSA due to the persistent burden of intermittent hypoxia. We also aimed to explore whether Panx1 concentrations correlate with polysomnographic severity indices, such as the apnea–hypopnea index (AHI) and oxygen desaturation parameters. By evaluating Panx1 across different OSA phenotypes, we sought to determine its potential utility as a diagnostic biomarker and its relationship with the severity of nocturnal hypoxemia.

## 2. Methods

### 2.1. Study Design and Participants

This cross-sectional study was conducted at the Sleep Laboratory of the Department of Chest Diseases, Süleyman Demirel University Faculty of Medicine. The study population consisted of 40 patients diagnosed with obstructive sleep apnea (OSA) and 40 symptomatic individuals referred for PSG who did not meet diagnostic criteria for OSA. All participants were aged 18 years or older and underwent overnight polysomnography (PSG) for diagnostic evaluation. OSA diagnosis and severity classification were established based on the American Academy of Sleep Medicine (AASM) criteria [9,10,11]. Individuals with chronic inflammatory conditions, malignancies, cardiovascular diseases, or those receiving antioxidant or anti-inflammatory treatments were excluded to minimize confounding factors.

### 2.2. Polysomnography

Standard overnight PSG was performed using a digital sleep monitoring system. Recorded parameters included electroencephalography, electrooculography, electromyography, electrocardiography, nasal and oral airflow, thoracoabdominal respiratory effort, and oxygen saturation. Key indices such as the apnea–hypopnea index (AHI), oxygen desaturation index (ODI), mean oxygen saturation (mean SpO_2_), minimum oxygen saturation (min SpO_2_), and the percentage of total sleep time with oxygen saturation below 90% (T90) were automatically calculated by the system. OSA severity was categorized as mild (5 ≤ AHI < 15), moderate (15 ≤ AHI < 30), and severe (AHI ≥ 30) according to AASM guidelines [10,11].

### 2.3. Biochemical Analysis

Venous blood samples were collected from all participants in the morning following PSG, after an overnight fast. Serum was separated by centrifugation and stored at −80 °C until analysis. Serum Panx1 concentrations were quantified using a commercially available enzyme-linked immunosorbent assay (ELISA) kit, following the manufacturer’s protocol. All samples and standards were analyzed in duplicate to ensure accuracy, and concentrations were determined based on a standard curve generated from known concentrations.

### 2.4. Statistical Analysis

Continuous variables were expressed as mean ± standard deviation (SD) or median with interquartile range (IQR), depending on data distribution assessed by the Shapiro–Wilk test. Categorical variables were presented as counts and percentages. Group comparisons for continuous variables were performed using Welch’s t-test or the Mann–Whitney U test, as appropriate. Categorical variables were compared using the chi-square test or Fisher’s exact test when expected frequencies were low. Standardized mean differences (SMD) were calculated to quantify the magnitude of between-group differences, and effect size was further assessed using Cohen’s d for serum Panx1 levels. Multivariable logistic regression models were constructed to evaluate the independent association between serum Panx1 levels and OSA status, adjusting for potential confounders including age, sex, and body mass index (BMI). Model calibration was assessed using the Hosmer–Lemeshow goodness-of-fit test. To further address potential confounding due to baseline differences between groups, an additional propensity score–matched sensitivity analysis was performed. Propensity scores were estimated using a logistic regression model incorporating age, sex, and BMI. One-to-one nearest-neighbor matching without replacement was applied. Post-matching analyses were conducted to assess whether the association between serum Panx1 levels and OSA status remained consistent after balancing major baseline covariates. The diagnostic performance of Panx1, both alone and combined with clinical variables, was assessed using receiver operating characteristic (ROC) curve analysis, with area under the curve (AUC) values reported. Differences in serum Panx1 levels across OSA phenotypes (Positional, REM-related, and Non-positional OSA) were evaluated as an exploratory analysis. Due to the small sample size in certain subgroups (e.g., R-OSA), non-parametric Kruskal–Wallis tests followed by Dunn’s post hoc tests were employed to minimize the impact of outliers and non-normal distributions. All statistical tests were two-sided, and a *p* value < 0.05 was considered statistically significant. Statistical analyses were performed using SPSS version 29 (IBM Corp., Armonk, NY, USA).

### 2.5. Ethics Statement

The study protocol was approved by the Clinical Research Ethics Committee of Süleyman Demirel University (Approval number: 22.06.2023/136). Written informed consent was obtained from all participants. All methods were performed in accordance with the relevant guidelines and regulations and the Declaration of Helsinki.

### 2.6. AI-Assisted Language Editing

ChatGPT (OpenAI, GPT-5.5) was used exclusively for English language refinement and editorial improvement during manuscript preparation. No AI tools were used for study design, data generation, statistical analysis, interpretation of results, or scientific decision-making. All outputs were critically reviewed by the authors.

## 3. Results

A total of 80 subjects were included in the study: 52 (65%) males and 28 (35%) females, with a male-to-female ratio of 1.86. The OSA group consisted predominantly of males compared to the control group (*p* = 0.004). The mean age of patients in the OSA group (52.63 ± 12.36 years) was significantly higher than that of the control group (46.00 ± 12.43 years; *p* = 0.019). Mean BMI was 33.15 ± 6.83 kg/m^2^ in the OSA group and 27.23 ± 4.88 kg/m^2^ in controls (*p* < 0.001). There was no significant difference in smoking status between groups (*p* > 0.05) (Table 1).

Regarding polysomnographic parameters, mean AHI and ODI were significantly higher in the OSA group compared to controls (37.65 ± 22.94 vs. 2.96 ± 1.27 events/hour and 34.27 ± 22.90 vs. 2.97 ± 1.74 events/hour, respectively; both *p* < 0.001). Mean oxygen saturation during sleep was lower in the OSA group (89.58 ± 3.55%) than in controls (93.93 ± 1.52%; *p* < 0.001). Minimum oxygen saturation and duration below 90% (T90) were also significantly worse in the OSA group (*p* < 0.001 for both) (Table 1). Serum Panx1 concentrations were significantly lower in patients with OSA (4.27 ± 2.66 ng/mL) than in controls (6.24 ± 4.75 ng/mL; *p* = 0.013) (Table 1).

Standardized mean differences (SMD) were calculated for all continuous variables to quantify the magnitude of between-group differences, with larger absolute SMD values indicating greater separation between the OSA and control groups. All continuous variables exceeded this threshold, confirming a pronounced separation between the OSA (Group 1) and control (Group 2) groups. Among polysomnographic parameters, the most substantial imbalances were observed for minimum oxygen saturation (MOS; SMD = −2.24), AHI (SMD = 2.14), ODI (SMD = 1.93), oxygen saturation (OS; SMD = −1.59), and T90 (SMD = 1.59), all reflecting very large effect sizes. These findings are consistent with the pathophysiological hallmarks of OSA, characterized by recurrent apneic events, oxygen desaturation, and prolonged nocturnal hypoxemia. Regarding anthropometric and demographic variables, BMI demonstrated a large imbalance (SMD = 1.00), with OSA patients being substantially more obese than controls. Age showed a moderate imbalance (SMD = 0.53), with OSA patients being older on average. Serum Panx1 levels also exhibited a moderate imbalance (SMD = −0.51), with lower concentrations observed in the OSA group compared to controls, suggesting a potential association between Panx1 and OSA-related pathophysiology.

Collectively, these SMD values confirm that the OSA and control groups are clinically and statistically well differentiated across all key parameters, supporting the validity of the group classification used in this study (Table 1).

### 3.1. Pannexin-1 Analysis

**Figure 1:**
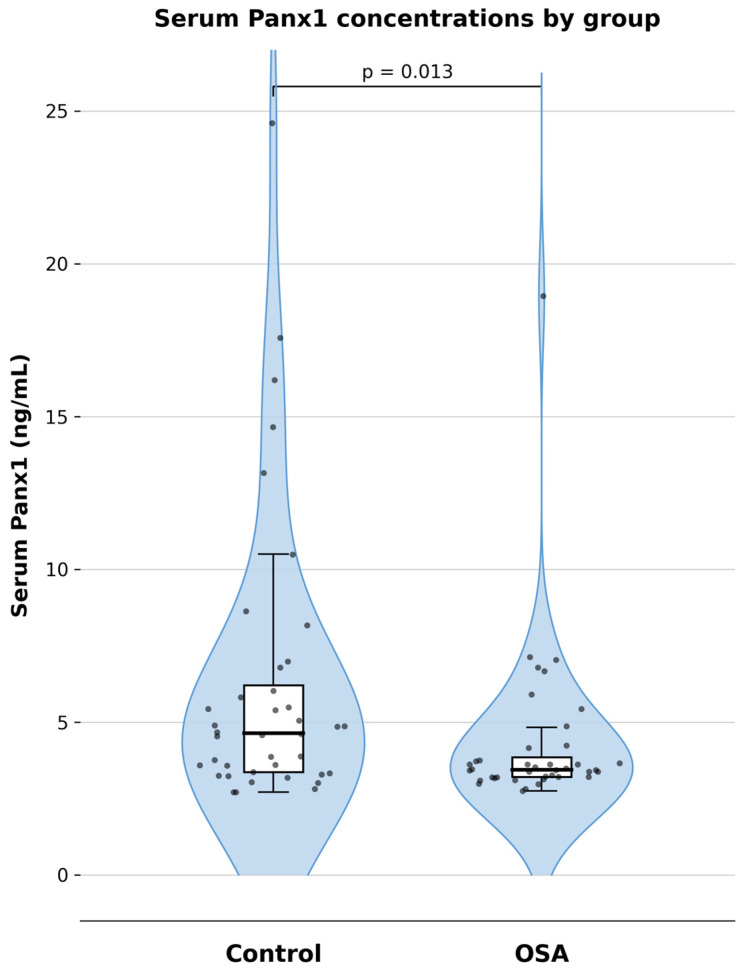
Serum Panx1 concentrations by group. A combined violin, box, and swarm plot illustrates the distribution of serum Panx1 levels in the control and OSA groups. The violin plot (shaded area) depicts the kernel density estimate, reflecting the probability distribution of Panx1 values across their range. The central box plot indicates the median and interquartile range (IQR), with whiskers extending to 1.5 × IQR. The overlaid swarm plot (individual black dots) displays each participant’s data point to visualize within-group variability and overlap. Serum Panx1 levels were significantly lower in the OSA group compared with the control group (Mann–Whitney *U* test, *p* = 0.013). Abbreviations: IQR, interquartile range; OSA, obstructive sleep apnea; Panx1, pannexin-1.

The distribution of serum Panx1 concentrations is illustrated using a combination of violin, box, and swarm plots to provide a comprehensive visualization of the data. The violin plot (shaded area) depicts the kernel density estimate, reflecting the probability distribution of Panx1 values across their range. The central box plot indicates the median and interquartile range (IQR), while the swarm plot (individual black dots) overlays each participant’s data point to display the degree of overlap and dispersion within and between groups. Serum Panx1 levels were significantly lower in the OSA group compared with the control group (Mann–Whitney U test, *p* = 0.013). The between-group difference corresponded to a Cohen’s *d* of 0.51, indicating a medium effect size (Table 1) (Figure 1).

Serum Panx1 concentrations differed across control subjects and OSA severity categories (Kruskal–Wallis, *p* = 0.0098). Descriptively, Panx1 levels were lower in the moderate and severe OSA groups compared with controls, whereas the mild OSA subgroup showed higher values; however, the mild OSA sample size was small (*n* = 5), limiting precision. In multiple-comparison post-hoc analyses using Mann–Whitney U tests with Holm adjustment, none of the pairwise differences remained statistically significant (all adjusted *p* ≥ 0.083), and Panx1 levels were similar between moderate and severe OSA (adjusted *p* = 0.893). Overall, these results suggest an association between Panx1 and OSA severity at the global level, but do not provide strong evidence for a clear stepwise dose–response pattern across severity strata in this cohort, particularly given the limited mild OSA sample.

Within-group analyses showed no evidence that serum Panx1 concentrations differed by sex or current smoking status in either the OSA or control group. In the OSA group, Panx1 levels were similar between males and females (Mann–Whitney U, *p* = 0.919; Cohen’s *d* = 0.20) and between current smokers and non-smokers (*p* = 0.934; *d* = 0.27). Likewise, in the control group, Panx1 concentrations did not significantly differ by sex (*p* = 0.871; *d* = −0.30) or smoking status (*p* = 0.161; *d* = 0.36). Overall, these findings suggest that the observed case–control difference in serum Panx1 is unlikely to be explained solely by sex distribution or current smoking in this cohort, although the female sample size in the OSA group was limited, which may reduce power to detect small subgroup effects.

In subgroup analyses stratified by diagnosis, correlations between serum Panx1 concentrations and polysomnographic parameters differed between the OSA and control groups. In the OSA group (Group 1, *n* = 40), serum Panx1 levels showed modest, inverse correlations with AHI (ρ = −0.32, *p* = 0.043) and T90 (ρ = −0.33, *p* = 0.041), indicating that higher Panx1 concentrations were associated with a lower apnea–hypopnea burden and shorter cumulative time spent with SpO_2_ < 90%. The correlations with ODI (*r* = −0.27, *p* = 0.093) and OS (*r* = 0.21, *p* = 0.195) did not reach statistical significance but followed a similar directional trend.

In contrast, within the control group (Group 2, *n* = 40), serum Panx1 concentrations were not significantly correlated with age, BMI, or any polysomnographic indices, including AHI, ODI, T90, MOS, or OS (all *p* > 0.10). These findings suggest that the relationship between Panx1 and sleep-related breathing disturbances is more apparent in patients with established OSA, whereas such associations are absent or negligible in controls (Table 2).

Spearman rank correlation coefficients (ρ) were calculated separately in the OSA and control groups to assess the association between serum Panx1 concentrations and each parameter. *p* < 0.05 was considered statistically significant.

**Figure 2:**
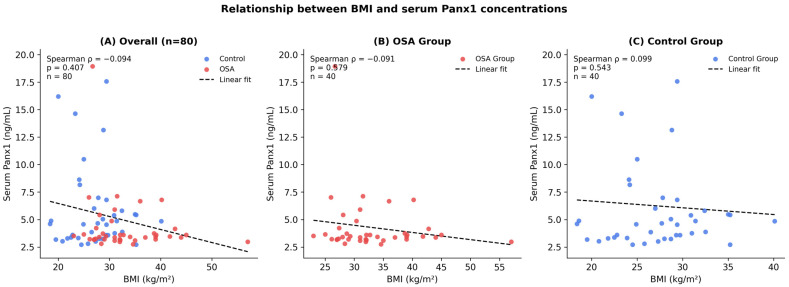
Relationship between body mass index (BMI) and serum pannexin-1 (Panx1) concentrations. (**A**) Overall cohort (*n* = 80); OSA patients (red) and controls (blue) are shown separately. (**B**) OSA group (*n* = 40). (**C**) Control group (*n* = 40). Dashed lines represent linear regression fits for visual reference. Spearman rank correlation coefficients (ρ) and corresponding *p*-values are displayed within each panel. *p* < 0.05 was considered statistically significant.

In the overall cohort (*n* = 80), serum Panx1 concentrations were not significantly associated with BMI. Spearman correlation analysis showed a weak inverse relationship that did not reach statistical significance (ρ = −0.094, *p* = 0.407), indicating no clear monotonic association between adiposity and circulating Panx1 levels in this dataset. When stratified by group, serum Panx1 concentrations were not significantly correlated with BMI in either the OSA or control group. In the OSA group (*n* = 40), Spearman correlation analysis revealed a negligible inverse association between BMI and serum Panx1 (ρ = −0.091, *p* = 0.579). Similarly, in the control group (*n* = 40), no significant correlation was observed (ρ = 0.099, *p* = 0.543), with the direction of the trend being weakly positive. These findings indicate that BMI does not independently explain the variability in serum Panx1 concentrations in either group, suggesting that the case–control difference in Panx1 levels is unlikely to be confounded by differences in adiposity (Figure 2).

**Figure 3:**
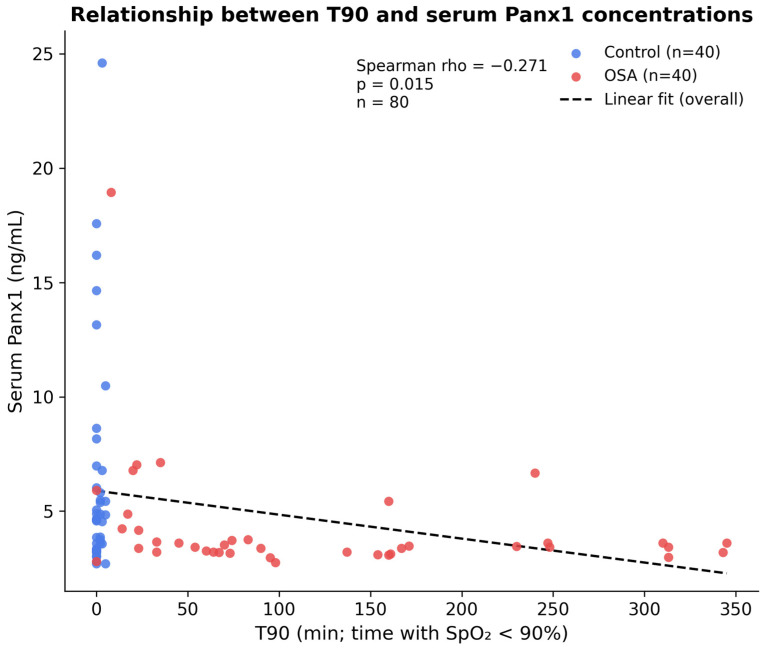
Relationship between the cumulative time spent with oxygen saturation below 90% (T90) and serum pannexin-1 (Panx1) concentrations. Scatter plots are shown for the overall cohort (*n* = 80) and stratified by group: OSA (red circles, *n* = 40) and control (blue circles, *n* = 40). Dashed lines represent linear regression fits for visual reference. Spearman rank correlation coefficients and corresponding *p*-values are displayed for the overall cohort. *p* < 0.05 was considered statistically significant. Abbreviations: OSA, obstructive sleep apnea; Panx1, pannexin-1; SpO_2_, oxygen saturation; T90, cumulative time with SpO_2_ below 90%.

In the overall cohort, higher T90 values were associated with lower serum Panx1 concentrations (Spearman ρ = −0.271, *p* = 0.015), indicating that prolonged nocturnal hypoxemia is modestly but significantly linked to reduced circulating Panx1. When stratified by group, this inverse relationship remained significant in the OSA group (ρ = −0.325, *p* = 0.041), whereas no significant association was observed among controls (ρ = 0.134, *p* = 0.410). These findings suggest that the relationship between T90 and Panx1 is driven primarily by patients with OSA and may reflect hypoxia-related alterations in Panx1 signaling in this population (Figure 3).

**Figure 4:**
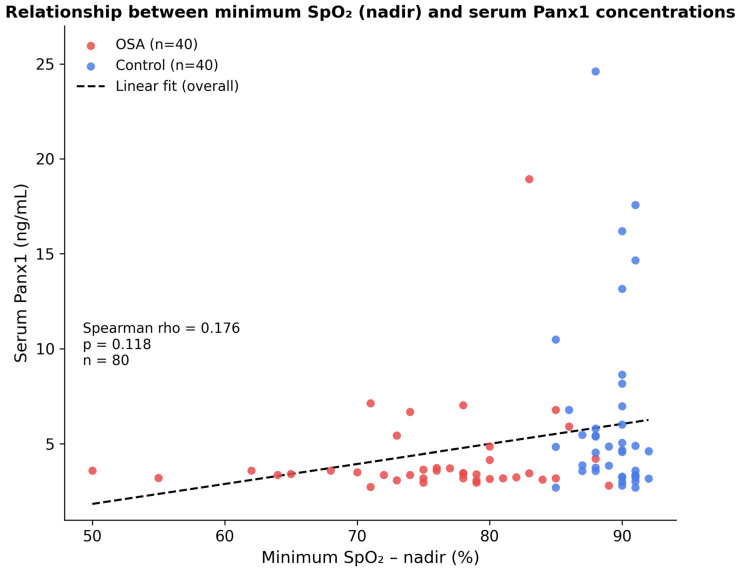
Relationship between minimum oxygen saturation (SpO_2_ nadir) and serum pannexin-1 (Panx1) concentrations. Scatter plots are shown for the overall cohort (*n* = 80), with OSA patients (red circles, *n* = 40) and controls (blue circles, *n* = 40) displayed separately. The dashed line represents the linear regression fit for the overall cohort. Spearman rank correlation coefficients and corresponding *p*-values are displayed for the overall cohort. *p* < 0.05 was considered statistically significant. Abbreviations: MOS, minimum oxygen saturation (nadir); OSA, obstructive sleep apnea; Panx1, pannexin-1; SpO_2_, oxygen saturation.

No significant association was observed between minimum SpO_2_ (nadir) and serum Panx1 concentrations in the overall cohort (Spearman rho = 0.176, *p* = 0.118). Subgroup analyses confirmed the absence of a significant correlation in both the OSA group (rho = 0.002, *p* = 0.990) and the control group (rho = −0.189, *p* = 0.242), (Figure 4). These findings suggest that the lowest nocturnal oxygen saturation value alone does not independently predict serum Panx1 levels in this cohort, in contrast to the cumulative hypoxic burden measured by T90, which was significantly associated with Panx1 concentrations. Notably, the association between T90 and Panx1 was more pronounced and remained significant within the OSA group (rho = −0.325, *p* = 0.041), whereas no such relationship was observed in the control group. These results suggest that the duration of exposure to nocturnal hypoxemia, rather than the absolute depth of the oxygen desaturation (nadir), is a more robust predictor of reduced circulating Panx1 levels in patients with OSA. This may indicate that chronic, prolonged hypoxic stress leads to a more substantial downregulation or depletion of Panx1 compared to transient, acute desaturation events.

### 3.2. Logistic Regression Models

Logistic regression analyses were performed to evaluate the association between serum Panx1 concentrations and OSA status across three models. In the unadjusted model (Model 1), lower serum Panx1 was associated with a trend toward increased odds of OSA (OR = 0.840, 95% CI: 0.705–1.001, *p* = 0.051), which did not reach conventional statistical significance. After adjustment for age and BMI (Model 2), the association was further attenuated (OR = 0.876, 95% CI: 0.728–1.055, *p* = 0.163). In the fully adjusted model (Model 3), which additionally included sex and smoking status, serum Panx1 remained a non-significant predictor of OSA (OR = 0.880, 95% CI: 0.713–1.088, *p* = 0.238). Notably, BMI (OR = 1.284, 95% CI: 1.113–1.483, *p* < 0.001) and male sex (OR = 11.755, 95% CI: 2.615–52.843, *p* = 0.001) were independently associated with OSA status in the fully adjusted model. These findings suggest that while serum Panx1 levels differ between OSA patients and controls, Panx1 does not independently predict OSA status after adjustment for established risk factors (Table 3). Importantly, the Hosmer–Lemeshow goodness-of-fit test demonstrated acceptable calibration of the fully adjusted model (χ^2^ = 12.91, df = 8, *p* = 0.115), supporting the adequacy of model performance.

### 3.3. ROC Curve Analysis for Logistic Regression Models Predicting OSA Status

**Figure 5:**
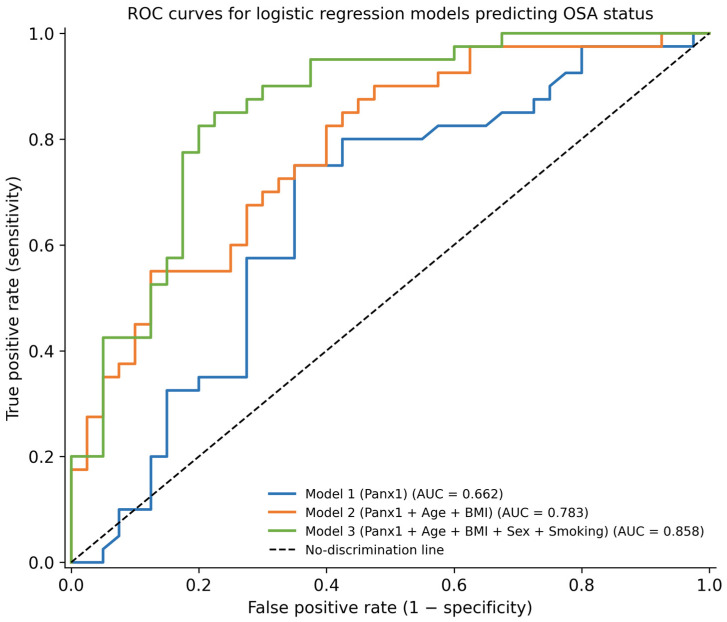
Receiver operating characteristic (ROC) curves for logistic regression models predicting OSA status. Model 1 (blue): serum Panx1 alone (AUC = 0.662; 95% CI: 0.537–0.783). Model 2 (orange): Panx1 + age + BMI (AUC = 0.783; 95% CI: 0.673–0.876). Model 3 (green): fully adjusted model including Panx1, age, BMI, sex, and smoking status (AUC = 0.858; 95% CI: 0.770–0.937). The diagonal dashed line represents the no-discrimination reference line. AUC values with 95% bootstrap confidence intervals are reported. Abbreviations: AUC, area under the receiver operating characteristic curve; BMI, body mass index; CI, confidence interval; OSA, obstructive sleep apnea; Panx1, pannexin-1; ROC, receiver operating characteristic.

ROC curve analysis was performed to evaluate the discriminative performance of the prediction models for OSA status. The unadjusted Panx1-only model demonstrated modest discrimination (AUC = 0.662; 95% bootstrap CI: 0.537–0.783). For serum Panx1 alone, ROC analysis identified an optimal cut-off value of 3.75 ng/mL using the Youden index, corresponding to a sensitivity of 75% and a specificity of 65% for discriminating OSA from control subjects. Adding established clinical covariates improved diagnostic accuracy: the model including Panx1, age, and BMI showed good discrimination (AUC = 0.783; 95% CI: 0.673–0.876), while the fully adjusted model (Panx1, age, BMI, sex, and smoking) achieved the highest performance (AUC = 0.858; 95% CI: 0.770–0.937). Using the Youden-index optimal threshold, the fully adjusted model provided a sensitivity of 0.825 and specificity of 0.800, indicating improved overall classification compared with the Panx1-only model. These findings suggest that Panx1 contributes limited standalone discrimination, whereas its predictive utility is maximized when incorporated into a multivariable risk model (Figure 5).

### 3.4. Sensitivity Analysis Using Propensity Score Matching

To further address potential confounding related to baseline imbalances between groups, an additional propensity score-matched sensitivity analysis was performed using age, sex, and BMI. One-to-one nearest-neighbor matching identified 25 matched pairs. After matching, demographic and anthropometric characteristics became more comparable between groups (Table 4). Serum Panx1 concentrations remained numerically lower in patients with OSA compared with controls (4.52 ± 3.28 vs. 5.63 ± 3.50 ng/mL); however, the difference was attenuated and no longer reached statistical significance (*p* = 0.25). These findings suggest that baseline demographic and anthropometric characteristics may partially contribute to the observed association between OSA and circulating Panx1 levels.

### 3.5. Exploratory Analysis of OSA Phenotypes

Serum Panx1 concentrations were compared across three OSA phenotypes: positional OSA (P-OSA), REM-related OSA (R-OSA), and non-positional OSA (Non-P-OSA). Mean Panx1 levels were similar in the P-OSA and Non-P-OSA groups (3.97 ± 1.40 ng/mL and 3.69 ± 0.93 ng/mL, respectively), with closely overlapping medians and interquartile ranges (P-OSA: 3.40 [3.19–3.85] ng/mL; Non-P-OSA: 3.45 [3.22–3.61] ng/mL). The overall between-group difference was not statistically significant (Kruskal–Wallis H(2) = 3.264, *p* = 0.196), with a small effect size (ε^2^ = 0.034). Post-hoc Dunn tests with Holm adjustment did not identify any significant pairwise differences (all adjusted *p* ≥ 0.236). However, the R-OSA group showed a numerically higher mean Panx1 level (8.10 ± 7.31 ng/mL) and a right-skewed distribution, as reflected by a median of 5.04 [3.96–9.17] ng/mL. Although the R-OSA phenotype appears to be associated with higher Panx1 concentrations, this subgroup comprised only four patients, and the large standard deviation and wide interquartile range indicate substantial intra-phenotype variability. Therefore, these findings should be interpreted cautiously and considered hypothesis-generating rather than definitive. Overall, Panx1 levels seem broadly comparable between positional and non-positional OSA phenotypes, while REM-related OSA may represent a distinct subgroup with potentially higher Panx1 levels that warrants further investigation in larger cohorts.

## 4. Discussion

Obstructive sleep apnea (OSA) is characterized by recurrent episodes of apnea and hypopnea accompanied by intermittent hypoxemia (IH), fluctuations in intrathoracic pressure, and sleep fragmentation resulting from inspiratory efforts against an occluded upper airway. Although arousals and intrathoracic pressure swings contribute to sympathetic activation and related comorbidities, IH is considered a major determinant of OSA-related morbidity and mortality. In the present study, lower circulating Panx1 levels were observed in patients with OSA and were inversely associated with markers of nocturnal hypoxemic burden, particularly T90. Although Panx1 alone demonstrated modest discriminatory ability for OSA, its integration with clinical variables substantially improved diagnostic performance. These findings suggest that Panx1 may reflect cumulative hypoxic stress in OSA and may contribute to multi-parameter biomarker approaches for risk stratification.

Two principal patterns of hypoxemia have been described in the literature. The key distinction between the short, high-frequency hypoxemia typically observed in OSA and the prolonged, low-frequency hypoxemia seen in chronic respiratory diseases lies in the reoxygenation cycles. The cyclical fluctuations of IH resemble ischemia–reperfusion injury and contribute to increased production of reactive oxygen species (ROS) and the development of oxidative stress [2]. IH also exerts significant effects on the vascular system through mechanisms such as systemic inflammation, increased endothelin release, and reduced nitric oxide (NO) production. Circulating NO levels, which promote pulmonary vasodilation, have been reported to be decreased in patients with OSA and to increase following positive airway pressure (PAP) therapy [12].

The earliest evidence suggesting that pannexin-1 (Panx1) channels may contribute to ischemic injury originated from studies demonstrating their activation in isolated hippocampal neurons under oxygen–glucose deprivation (OGD). In these experiments, a large component of delayed anoxic depolarization currents could be blocked by carbenoxolone and lanthanum, supporting a role for Panx1 channels in hypoxia-related neuronal injury [13]. Zhang et al. subsequently showed that cerebral ischemia facilitates Panx1-mediated calcein efflux under OGD conditions and proposed that increased levels of reactive nitrogen species, particularly nitric oxide (NO), promote channel activation through S-nitrosylation of intracellular cysteine residues [14]. In contrast, later studies demonstrated that S-nitrosylation of the C346 cysteine residue in response to NO donors inhibited Panx1 channel currents and ATP release, suggesting a potential negative feedback mechanism that limits excessive channel activation during oxidative stress [15,16]. These apparently conflicting findings indicate that the precise mechanisms by which oxidative and nitrosative stress regulate Panx1 function remain incompletely understood.

Clinically, elevated circulating Panx1 levels have been reported in patients with acute ischemic stroke compared with healthy controls, with a proposed cut-off value of ≥5.0 ng/mL predicting stroke with high sensitivity and specificity [17]. Together, these observations highlight the potential role of Panx1 as a mediator of hypoxia-related cellular stress and inflammatory signaling. Given that intermittent hypoxia represents a central pathophysiological feature of OSA, alterations in circulating Panx1 levels may reflect systemic responses to recurrent hypoxic stress in this population. One potential mechanistic explanation is that recurrent intermittent hypoxia in OSA may induce oxidative stress and ATP-mediated inflammatory signaling through Panx1 channel activation. Excess extracellular ATP release has been implicated in activation of purinergic pathways and downstream inflammatory cascades, including NLRP3 inflammasome signaling, which has previously been linked to hypoxia-related tissue injury and endothelial dysfunction [4,5]. Over time, chronic exposure to intermittent hypoxia may lead to adaptive alterations in Panx1 regulation or cellular stress responses, potentially contributing to the lower circulating concentrations observed in OSA.

Recent evidence suggests that pannexin-1 (Panx1) may also play a role in regulating cerebral homeostasis during the sleep–wake cycle through ATP release, downstream activation of adenosine receptors, and interactions with other somnogens [18]. In patients with chronic insomnia disorder, Su et al. reported significantly higher serum Panx1 concentrations compared with healthy controls, suggesting a potential relationship between circulating Panx1 levels and sleep-related neuronal stress or dysregulation [19]. Beyond neuronal injury, Panx1 channels represent an important pathway for non-vesicular ATP release, a process that may exhibit circadian variation and is closely linked to purinergic signaling pathways involved in sleep–wake regulation. Experimental studies have further suggested that pannexins may contribute to regulation of cerebral homeostasis and sleep–wake physiology through interactions with purinergic signaling pathways, raising the possibility that their activity may be influenced by endogenous biological rhythms [18,20].

Although direct evidence for circadian variation in circulating Panx1 levels in humans remains limited, existing findings raise the possibility that serum concentrations may be influenced by time-of-day effects and sleep–wake regulatory mechanisms. In the present study, blood samples were collected at a standardized morning time point following overnight polysomnography to minimize variability. However, reliance on a single post-awakening measurement limits the ability to distinguish stable biological alterations from transient effects related to acute nocturnal hypoxemia, sleep architecture, or physiological responses occurring during the preceding sleep period. Future studies incorporating serial sampling across multiple time points are needed to clarify the temporal dynamics of Panx1 and its interaction with intermittent hypoxemia in OSA.

To our knowledge, this study represents one of the first clinical investigations evaluating circulating pannexin-1 (Panx1) levels in patients with obstructive sleep apnea (OSA). Overall, lower serum Panx1 concentrations were observed in patients with OSA compared with non-OSA control subjects. In our cohort, Panx1 levels showed negative correlations with key polysomnographic parameters, including the apnea–hypopnea index (AHI), oxygen desaturation index (ODI), and the cumulative time spent with oxygen saturation below 90% (T90), suggesting a relationship between reduced Panx1 levels and increasing hypoxic burden.

Because body mass index (BMI) differed significantly between groups, we additionally explored the relationship between BMI and serum Panx1 levels to assess potential confounding effects. In scatter-plot analyses, BMI showed no significant correlation with Panx1 concentrations within either the OSA group or the control group, and only a weak, non-significant inverse trend was observed in the overall cohort. These findings suggest that the observed reduction in Panx1 levels in patients with OSA is unlikely to be solely explained by differences in adiposity. Nevertheless, residual confounding related to BMI or other metabolic factors cannot be entirely excluded and should be considered when interpreting the results.

To further address the potential influence of baseline imbalances, we performed a propensity score-matched sensitivity analysis using age, sex, and BMI. Following matching, the direction of the association between OSA status and lower serum Panx1 concentrations remained unchanged; however, the difference no longer reached statistical significance. These findings suggest that demographic and anthropometric characteristics may partially contribute to the observed association and further support a cautious interpretation of the findings.

Sex-related biological differences may also have influenced the observed findings. Although serum Panx1 concentrations did not significantly differ according to sex within our cohort, the predominance of male participants in the OSA group may limit the generalizability of these findings. In addition, the relatively small number of female participants reduced the statistical power for sex-specific comparisons. Future studies with more balanced sex distributions will be important to clarify potential sex-specific patterns in circulating Panx1 responses.

When patients were evaluated according to OSA phenotypes, the apnea–hypopnea index (AHI) was highest in the REM-related OSA (R-OSA) group. Although this phenotype exhibited the lowest mean oxygen saturation, the cumulative time spent with oxygen saturation below 90% (T90) was longer in the non-positional OSA (Non-P-OSA) group, indicating a greater hypoxemic burden. These differences were statistically significant (*p* < 0.001).

Regarding serum Panx1 concentrations, the lowest mean levels were observed in the Non-P-OSA group, whereas the highest levels were found in the R-OSA group. Descriptive differences in Panx1 concentrations across phenotypes were observed; however, statistical comparisons using the Kruskal–Wallis test followed by Dunn post hoc analyses with Holm adjustment did not identify significant between-group differences, and the overall effect size was small. These findings suggest that although phenotype-specific differences in Panx1 levels may exist, the current results should be interpreted cautiously and considered hypothesis-generating, particularly given the limited sample size within certain phenotype subgroups.

An intriguing observation in our study was the apparent paradox between hypoxemic burden and Panx1 concentrations across OSA phenotypes. Specifically, the non-positional OSA group exhibited greater hypoxemic burden yet lower serum Panx1 concentrations, whereas the REM-related OSA subgroup demonstrated the opposite pattern. One possible explanation may involve a biphasic response in which moderate intermittent hypoxemia triggers compensatory Panx1 release as part of purinergic stress signaling [8,21], whereas more prolonged or severe hypoxic exposure may lead to adaptive alterations in Panx1 regulation. In addition, REM-related respiratory events may produce greater oxygen desaturation and sympathetic activation despite relatively milder overall polysomnographic severity, potentially contributing to the relatively higher Panx1 concentrations observed in this subgroup [22]. However, these interpretations remain speculative and should be considered hypothesis-generating, particularly given the very small REM-related OSA subgroup and its substantial variability.

Our ROC and multivariable analyses further clarify the potential clinical relevance of Panx1 in OSA. Although serum Panx1 alone demonstrated only modest discriminatory performance (AUC = 0.662), its diagnostic utility improved substantially when combined with established clinical variables such as age, BMI, and sex, with the fully adjusted model achieving an AUC of approximately 0.86. These findings suggest that Panx1 is unlikely to function as a standalone diagnostic biomarker but may provide clinically relevant biological information as part of a multi-parameter approach. Therefore, incorporating Panx1 into integrated risk models may improve characterization of hypoxia-related pathophysiological processes and contribute to more refined OSA risk assessment.

This study has several limitations that should be considered when interpreting the findings.

First, the OSA and control groups were not initially matched for age, sex, and BMI, all of which are known to influence OSA severity and systemic inflammatory or oxidative stress pathways. Although multivariable adjustment and an additional propensity score–matched sensitivity analysis were performed to address this issue, residual confounding cannot be fully excluded. Notably, Panx1 did not remain an independent predictor of OSA after adjustment for these covariates, and the matched analysis attenuated the observed association. These findings suggest that baseline demographic and anthropometric differences may partly contribute to the relationship between OSA status and circulating Panx1 levels.

Second, the control group consisted of symptomatic individuals referred for polysomnography who were subsequently found not to meet diagnostic criteria for OSA, rather than completely healthy non-snoring subjects. Although this approach reflects routine clinical practice, it may have introduced additional heterogeneity within the control population. Furthermore, simple snoring itself has been associated with low-grade inflammation and early endothelial dysfunction and therefore may not represent a biologically neutral condition. Consequently, the use of this comparison group may have attenuated the observed differences in serum Panx1 concentrations between groups. Inclusion of a healthier non-snoring control population might have resulted in greater between-group separation and potentially strengthened the observed associations.

Third, the overall sample size was relatively modest, particularly within specific phenotype subgroups such as REM-related OSA and among female participants within the OSA cohort, limiting statistical power for subgroup analyses.

Fourth, serum Panx1 concentrations were measured at a single time point using morning blood samples obtained following overnight polysomnography, which precluded evaluation of potential circadian variation in circulating Panx1 levels. Therefore, it remains difficult to determine whether the observed differences represent stable biological alterations or transient effects related to acute nocturnal hypoxemia, sleep architecture, or sleep–wake-associated physiological changes.

Fifth, the cross-sectional design and lack of longitudinal follow-up prevented evaluation of temporal changes in serum Panx1 concentrations or their response to therapeutic interventions such as CPAP treatment. Future longitudinal studies assessing Panx1 dynamics before and after treatment may help clarify its potential utility as a marker of disease activity and therapeutic response.

Taken together, these findings suggest that circulating Panx1 reflects important aspects of the hypoxemic burden associated with OSA and may capture phenotype-related differences that are not fully explained by conventional polysomnographic indices alone. Within the broader landscape of OSA biomarkers, Panx1 therefore represents a biologically plausible candidate that may contribute to improved characterization of hypoxia-related pathophysiology. However, further validation in larger and well-characterized cohorts is required to clarify its potential clinical utility.

### Clinical Implications

The present findings suggest several potentially relevant clinical implications. First, serum Panx1 appears to reflect cumulative nocturnal hypoxemia rather than isolated desaturation events, as supported by its association with T90 rather than minimum SpO_2_. Second, Panx1 demonstrated greater discriminatory performance when incorporated into a multi-parameter model alongside established clinical risk factors, rather than as a standalone biomarker. Finally, exploratory analyses across OSA phenotypes suggested possible differences in Panx1 concentrations according to hypoxic burden patterns; however, these observations should be interpreted cautiously given the limited subgroup sample sizes. Collectively, these findings support the potential role of Panx1 as a biologically plausible adjunctive biomarker that may contribute to improved characterization of OSA-related hypoxic burden and phenotypic heterogeneity.

## 5. Conclusions

This study demonstrates an association between lower circulating Pannexin-1 (Panx1) concentrations, OSA status, and nocturnal hypoxemic burden. Although serum Panx1 levels were lower in patients with OSA and showed modest discriminatory ability as a standalone marker, diagnostic performance improved when Panx1 was incorporated into multivariable models including established clinical factors. These findings support the potential role of Panx1 as a biologically plausible adjunct biomarker reflecting hypoxic burden rather than an independent diagnostic marker of OSA.

Exploratory analyses further suggested possible differences in Panx1 concentrations across OSA phenotypes, highlighting the complex interaction between intermittent hypoxia and purinergic signaling pathways. However, the observed associations appeared sensitive to baseline demographic characteristics and should therefore be interpreted cautiously.

Further studies involving larger, matched, and longitudinal cohorts are needed to validate these findings and clarify the mechanistic and potential clinical significance of Panx1 in OSA.

## Figures and Tables

**Table 1:** Baseline characteristics of the study population by group. Continuous variables are summarized as mean ± SD. Categorical variables are presented as *n* (%). *p*-values were obtained using ^a^ Mann–Whitney U test for continuous variables and ^b^ chi-square or Fisher’s exact test for categorical variables. Standardized mean differences (SMD) > 0.1 suggest imbalance between groups.

Variable	OSA Group	Control Group	Mean Difference (OSA–Control)	SMD	*p*-Value
Male, *n* (%)	32 (80.0)	20 (50.0)	30.0 pp		0.004 ^b^
Age (years)	52.62 ± 12.36	46.00 ± 12.43	6.62	0.53	0.019 ^a^
BMI (kg/m^2^)	33.15 ± 6.83	27.23 ± 4.88	5.92	1.00	<0.001 ^a^
Serum Panx1 (ng/mL)	4.27 ± 2.66	6.24 ± 4.75	−1.97	−0.51	0.013 ^a^
AHI (events/h)	37.64 ± 22.94	2.96 ± 1.27	34.68	2.14	<0.001 ^a^
ODI (events/h)	34.27 ± 22.90	2.97 ± 1.74	31.30	1.93	<0.001 ^a^
T90 (min; SpO_2_ < 90%)	120.00 ±105.59	1.15 ± 1.69	118.85	1.59	<0.001 ^a^
Minimum SpO_2_ (MOS, %)	75.78 ± 8.26	89.22 ± 1.90	−13.45	−2.24	<0.001 ^a^
O_2_ saturation (OS, %)	89.58 ± 3.55	93.92 ± 1.51	−4.35	−1.59	<0.001 ^a^
Current smoking, n (%)	16 (40.0)	13 (32.5)	7.5 pp		0.642 ^b^

Abbreviations: AHI, apnea–hypopnea index; BMI, body mass index; MOS, minimum oxygen saturation (nadir); ODI, oxygen desaturation index; OSA, obstructive sleep apnea; Panx1, pannexin-1; SMD, standardized mean difference; pp, percentage points.

**Table 2:** Correlations between serum Panx1 concentrations and demographic or polysomnographic parameters.

	Variable	Correlation Coefficient (ρ)	*p* Value
Group 1 OSA group	Age (years)	0.009	0.957
	BMI (kg/m^2^)	−0.091	0.579
	AHI (events/h)	−0.321	0.043
	ODI (events/h)	−0.269	0.093
	T90 (min; SpO_2_ < 90%)	−0.325	0.041
	Minimum SpO_2_ (MOS, %)	0.002	0.990
	OS (%)	0.209	0.195
Group 2 Control Group	Age (years)	−0.025	0.877
	BMI (kg/m^2^)	0.099	0.543
	AHI (events/h)	0.027	0.868
	ODI (events/h)	−0.012	0.940
	T90 (min; SpO_2_ < 90%)	0.134	0.410
	Minimum SpO_2_ (MOS, %)	−0.189	0.242
	OS (%)	−0.250	0.120

**Table 3:** Logistic regression analysis evaluating the association between serum Panx1 and OSA status. Abbreviations: BMI, body mass index; CI, confidence interval; OR, odds ratio; OSA, obstructive sleep apnea; Panx1, pannexin-1.

Model/Variable	OR	95% CI	*p*-Value
Model 1: Unadjusted–Serum Panx1	0.840	0.705–1.001	0.051
Model 2: Adjusted (Age, BMI)–Serum Panx1	0.876	0.728–1.055	0.163
Model 3: Fully adjusted–Serum Panx1	0.880	0.713–1.088	0.238
Model 3: Age	1.007	0.954–1.063	0.796
Model 3: BMI	1.284	1.113–1.483	<0.001
Model 3: Sex (male)	11.755	2.615–52.843	0.001
Model 3: Smoking	1.481	0.431–5.083	0.533

**Table 4:** Baseline characteristics after propensity score matching.

Variable	OSA Matched (*n* = 25)	Control Matched (*n* = 25)	*p*-Value
Male sex, *n* (%)	19 (76.0)	19 (76.0)	1.000
Age (years)	49.56 ± 11.11	47.28 ± 13.31	0.52
BMI (kg/m^2^)	32.18 ± 5.74	28.00 ± 5.34	0.07
Serum Panx1 (ng/mL)	4.52 ± 3.28	5.63 ± 3.50	0.25

## Data Availability

The datasets generated and analyzed during the current study contain sensitive personal health information and are therefore not publicly available due to restrictions imposed by national data protection regulations (Law on the Protection of Personal Data No. 6698, Türkiye). De-identified data may be provided by the corresponding author upon reasonable request and with appropriate institutional approval.
